# Genotype distribution of human papillomavirus (HPV) and co-infections in cervical cytologic specimens from two outpatient gynecological clinics in a region of southeast Spain

**DOI:** 10.1186/1471-2334-9-124

**Published:** 2009-08-10

**Authors:** Pablo Conesa-Zamora, Sebastián Ortiz-Reina, Joaquín Moya-Biosca, Asunción Doménech-Peris, Francisco Javier Orantes-Casado, Miguel Pérez-Guillermo, Marcos Egea-Cortines

**Affiliations:** 1 Grupo de Patología Molecular y Farmacogenética FFIS011, Hospital Universitario Santa María del Rosell, Cartagena, Spain; 2 Servicio de Anatomía Patológica, Hospital Universitario Santa María del Rosell, Spain; 3 Servicio de Análisis Clínicos, Hospital Universitario Santa María del Rosell, Spain; 4 Área de Genética, Instituto de Biotecnología Vegetal, Escuela Técnica Superior de Ingeniería Agronómica, Universidad Politécnica de Cartagena, Spain

## Abstract

**Background:**

Human Papillomavirus (HPV) genotype distribution and co-infection occurrence was studied in cervical cytologic specimens from Murcia Region, (southeast Spain), to obtain information regarding the possible effect of the ongoing vaccination campaign against HPV16 and HPV18.

**Methods:**

A total of 458 cytologic specimens were obtained from two outpatient gynecological clinics. These included 288 normal benign (N/B) specimens, 56 atypical squamous cell of undetermined significance (ASC-US), 75 low-grade squamous intraepithelial lesions (LSIL) and 39 high-grade squamous intraepithelial lesions (HSIL). HPV genotyping was performed using PCR and tube array hybridization.

**Results:**

The most frequent genotype found was HPV16 (14.9% in N/B; 17.9% in ASC-US; 29.3% in LSIL and 33.3% HSIL). Distribution of other genotypes was heavily dependent on the cytologic diagnoses. Co-infections were found in 15.3% of N/B, 10.7% of ASC-US, 48% of LSIL and 25.6% of HSIL cases (significantly different at p < 0.001). Strikingly, in N/B diagnoses, genotypes from A5 species were found as coinfecting in all cases. Genotypes from A7 or A9 species appeared in co-infections in 56.5% and 54% respectively whereas genotypes from A6 species appeared in 25.1% of cases.

**Conclusion:**

HPV vaccination might prevent 34.6% and 35.8% of LSIL and HSIL, respectively. Co-infection rate is dependent on both cytologic diagnosis and HPV genotype. Moreover, genotypes belonging to A5, A7 and A9 species are more often found as co-infections than genotype pertaining to A6 species. This suggests that phylogenetically related genotypes might have in common similar grades of dependency for cervical epithelium colonization.

## Background

It is well established that human papilloma virus (HPV) is necessary to develop cervical cancer [1]. There are more than one hundred HPV genotypes that can be phylogenetically ordered in several genus, species and types according to sequence similarity [2,3]. Importantly, several HPVs are known because they have strong oncogenic potential and are classified as high-risk HPVs [4]. Amongst the high-risk genotypes, HPV16 and 18 account for nearly 70% of cervical cancer cases worldwide [5,6]. In a substantial percentage of HPV infections, ranging from 20–30%, and depending on the HPV typing assay used, two or more different HPV genotypes may be found [7-10]. These co-infections seem to have a higher rate of occurrence than expected by chance [10,11] suggesting a synergy amongst the coinfecting HPV genotypes. However, whether certain types of HPV are more or less likely to be acquired together still remains unknown [11].

In order to prevent cervical carcinoma, two types of prophylactic vaccines have been developed: a bivalent vaccine against the high-risk HPV16 and 18 [12], and a tetravalent vaccine against HPV16/18, and HPV 6/11 two low-risk genotypes that are prevalently responsible for genital warts [13]. Both are based on virus-like particles containing L1 capsid protein of HPV [14].

The HPV genotypes found in different regions of the world vary both in type and relative incidence. There is also evidence of the prevalence of specific variants of defined genotypes in certain ethnic groups [15-17]. In fact, some HPV variants correlate with human migration flows [18], suggesting a degree of genotype fixation in certain populations. Therefore, the HPV vaccines could have an impact on the particular HPV genotype distribution of the region where the campaign is going to be launched. Several studies have demonstrated that these vaccines seem to protect against genotypes different from HPV16, 18, 6 and 11 [12,19-21], but the clinical relevance remains to be determined. As well as the presumed cross-protection effect, the decreasing prevalence of the genotypes included in the HPV vaccine may affect the prevalence of other coinfecting genotypes due to the possible interplay between them in the cervical epithelium. As suggested by Woodman et al. if different HPV genotypes compete to colonize the cervical epithelium the prevalence of the genotypes not targeted by the vaccines could increase, changing the genotype-associated risks [14]. These arguments suggest that the impact of the vaccine could vary depending on the distribution of regional HPV types and the coinfecting genotype patterns. The aim of the present work is to assess the local prevalence of HPV genotypes as single-type infection or as co-infection in female patients followed-up because of abnormal Pap smears as a starting point to understand the evolution of HPV types when vaccination becomes a mainstream health policy.

The Region of Murcia, located in the southeast of Spain, has a population of 1.4 millon and is currently divided into five administrative health areas. In the last ten years, a high influx of immigrants from northern Africa and Latin America has caused population increase of nearly ten percent. Regional health authorities have launched a campaign in 2008 to vaccinate all young females (aged 11 to 14 years old) against HPV16 and HPV18, thus making the Region of Murcia a good model system to study the effect of vaccination in HPV-related diseases.

## Methods

### Samples

Four hundred and fifty eight cytologic consecutive specimens collected from November 2006 to April 2009 were included in this study. Cytologic specimens correspond to single patients (median age 38 (SD = 10.0); range 19 to 68) referred to the outpatient gynecological clinics of Hospital Universitario Santa María del Rosell (HUSMR) and Hospital Rafael Méndez (HRM) because of previous abnormal Pap smears. These two hospitals provide healthcare to a population of about 535.000 individuals (38.1% of population of Murcia Region). The incidence of cervical cancer in Murcia region is 9/100,000. Smears were stained with Papanicolaou stain. A second sample was taken by gynecologists either with cotton swab or with cytobrush and resuspended in 400 μl of PBS for immediate DNA extraction and HPV genotyping. Papanicolaou smears were reviewed by a pathologist trained in gynecologic pathology (SOR). The cytologic diagnoses of the cases included in this study were as follows: 288 normal or benign (N/B) cytologic diagnoses, 56 cases with atypical squamous cell of undetermined significance (ASC-US), 75 cases with low-grade squamous intraepithelial lesions (LSIL), 39 cases with high-grade squamous intraepithelial lesions (HSIL). Informed consent was not required for this study since the results presented here come from HPV genotyping routinely performed as an adjunct to Pap smears in pathology department. In any case all identifiers were deleted in order to protect patient confidentiality. The study was approved by the local ethical board.

### Viral genotype determination

DNA extraction was performed from all samples using Maxwell 16 Cell DNA Purification Kit (#AS1020 Promega, Madison, USA) and Maxwell 16 automated DNA extraction station (Promega, Madison, USA) according to the instruction manual. Average DNA concentration in these samples was 60 ng/μl. Genotyping was performed with Clart HPV 2 kit (ClonDiag, Jena, Germany) [22,23]. Amplification of L1 conserved region was performed using biotin-labeled primers, and amplicons were denaturalized and hybridized on a low-density tube array containing HPV type-specific probes. HPV types genotyped with the kit included high-risk (HR) genotypes 16, 18, 31, 33, 35, 39, 45, 51, 52, 56, 58, 59, 68, 73 and 82; low-risk (LR) genotypes 6, 11, 40, 42, 43, 44, 54, 61, 66, 70, 72, 81 and 89 and genotypes with probable high-risk (PHR) 20, 53 and 66. This kit is CE labelled and has been validated in several studies [22,24]. Its analytical sensitivity is between 10–10^3 ^viral copies and diagnostic sensitivity and specificity for each genotype were 98.2% and 100%, respectively. A hybridization positive control containing biotin, a CFRT human gene internal control and HPV negative and positive controls were included in each run.

Genotypes were classified according to their cervical carcinoma associated risk as high, low and probable high-risk as reported by Muñoz et al [25]. The most frequent genotypes found in N/B cases were grouped in HPV species for co-infection analysis according to the phylogenetic classification proposed by de Villiers et al. [2]. A5 (HPV51 and 82), A6 (HPV53, 66 and 56), A7 (HPV18, 39, 45 and 68) and A9 (HPV16, 58, 31, 52, 33 and 35).

### Statistical analysis

HPV genotype distribution analysis was performed using SPSS.14.0 (Chicago, IL). χ^2 ^was used to evaluate statistical significance between grade of lesion (normal/benign, ASC-US, LSIL and HSIL) and HPV genotypes found in the study.

## Results

### Cytologic diagnoses and HPV presence

HPV genotyping was successfully achieved in all samples. The percentage of samples positive for an HPV infection increased with the severity of the lesion, from N/B (49.7%) to LSIL (82.7%) and HSIL (87.2%) (p < 0.0001) (Table 1). The occurrence of low versus high-risk HPV genotypes also differed between the distinct diagnoses (p < 0.05; Table 1).

**Table 1:** Association amongst cytologic, HPV positivity and HPV-associated risks.

Lesion	No. cases	HPV positivity n(%)	HR-HPV n(%)	LR-HPV n (%)	PHR-HPV n (%)
N/B	288	143 (49.7)	95 (33.0)	43 (14.9)	29 (10.1)
ASC-US	56	30 (53.6)	22 (39.3)	4 (7.1)	5 (8.9)
LSIL	75	62 (82.7)	53 (70.7)	17 (22.7)	16 (21.3)
HSIL	39	34 (87.2)	33 (84.6)	3 (7.7)	2 (5.1)

Total	458	269	203	67	52

HPV: Human Papilloma Virus.

HR: High-risk.

PHR: Probable high-risk.

LR: Low-risk.

N/B: normal/benign.

ASC-US: Atypical squamous cell of undetermined significance.

LSIL: Low-grade squamous intraepithelial lesion.

HSIL: High-grade squamous intraepithelial lesion.

### HPV genotype distribution changes in cytologic diagnoses

**Figure 1:**
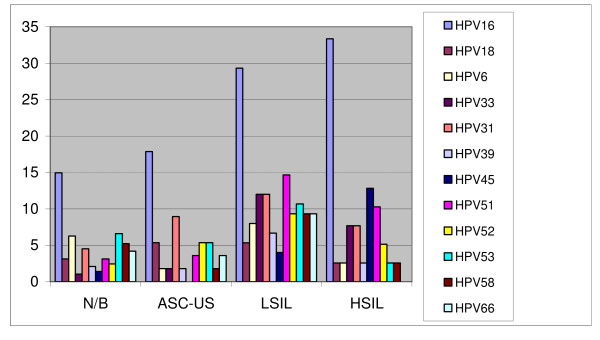
**Distribution of genotypes found in ASC-US, LSIL, and HSIL**. The most common genotypes are depicted in this bar chart. X axis: cytologic diagnoses. Y axis: relative genotype frequency (%) for each cytologic diagnosis.

The specific HPV genotypes changed depending on cytologic diagnoses (Figure 1). Independently of the diagnoses performed, HPV16 was the most frequent genotype. The most frequent genotypes in N/B cases were HPV16 (14.9%), HPV53 (6.6%), low-risk HPV6 (6.6%) and HPV58 (5.2%). In ASC-US the distribution was HPV16 (17.9%), HPV31 (4.5%) and three genotypes i.e HPV 18, 52 and 53 were detected in 5.2% to 5.4% of this lesion. In LSIL specimens HPV16, 51, 31 and HPV33 were observed in 29.3%, 14.7%, 12% and 12% of cases, respectively. Finally, in HSIL HPV distribution was HPV16 (33.3%), HPV45 (12.9%) and HPV51 (10.3%). HPV16 prevalence was significantly associated with increased grade of lesion (p < 0.005)

### Genotypes found as co-infections

**Figure 2:**
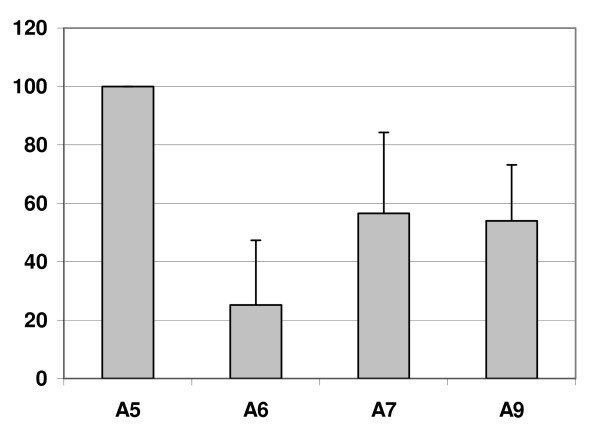
**Frequency of co-infection according to HPV species**. Most common genotypes were grouped in four species (X axis): A5 (HPV51 and 82), A6 (HPV53, 66 and 56), A7 (HPV18, 39, 45 and 68) and A9 (HPV16, 58, 31, 52, 33 and 35). Standard deviation is plotted for each species. Y axis: percentage of co-infection.

Significant differences in percentage of co-infection occurrence were found depending on cytologic diagnoses (Table 2) (p < 0.0001). Most co-infections consisted of two different genotypes, although in three samples up to seven different genotypes were found (one in N/B and two in LSIL; Table 2). The percentages of co-infections encompassing most frequent genotypes found in N/B cases are listed in table 3. Using the phylogenetic classification for HPV species we observed that in N/B specimens HPV genotypes belonging to the A5 family (HPV51 and 82) were always present as co-infections. This percentage was still high for A7 and A9 species where co-infection percentages were 56.5% and 54% respectively (Figure 2).

**Table 2:** Cytologic diagnoses and co-infection occurrence.

Co-infection	≥2 g types (%)	2 types	3 types	4 types	5 types	6 types	7 types
NB	44 (15.3)	24	15	3	1	0	1
ASCUS	6 (10.7)	4	2	0	0	0	0
LSIL	36 (48.0)	21	6	6	1	0	2
HSIL	10 (25.6)	7	1	0	2	0	0
Total	96 (100)*	56 (85.3)*	24 (25)*	9 (9.4)*	4 (4.2)*	0	3 (3.1)*

* Percentage referred to total of co-infections (n = 96).

N/B: normal or benign cases.

ASC-US: atypical squamous cell of undetermined significance.

LSIL: low-grade squamous intraepithelial lesions.

HSIL: high-grade squamous intraepithelial lesions.

**Table 3:** HPV genotypes found as single infection or co-infection in normal/benign cytologic specimens.

HPV Genotype	Risk	No. of cases	Single-type infections	Co-infections (%)*	Species
16	HR	43	25	18 (41.9)	A9
53	PHR	19	11	8 (42.1)	A6
6	LR	18	9	9 (50.0)	A10
58	HR	15	10	5 (33.3)	A9
31	HR	13	4	9 (69.2)	A9
66	PHR	12	8	4 (33.3)	A6
18	HR	9	2	7 (77.8)	A7
51	HR	9	0	9 (100)	A5
61	LR	9	3	6 (66.7)	A3
52	HR	7	2	5 (71.4)	A9
39	HR	6	2	4 (66.7)	A7
82	HR	5	0	5 (100)	A5
56	HR	5	5	0	A6
45	HR	4	3	1 (25)	A7
81	LR	4	1	3 (75)	A3

HPV: Human Papilloma Virus.

HR: High-risk.

PHR: Probable high-risk.

LR: Low-risk.

Co-infections: Number of cases in which the given genotype is found with one or more different HPV genotype/s in the same cytologic specimen.

*Percentage referred to the total number of infections for a given genotype.

## Discussion

In the present study we have found an HPV detection rate of 49.7% in N/B cases, 53.6% in ASCUS, 82.7% in LSIL and 87.2% in HSIL. The N/B percentage is considerably higher than that found in other studies with Spanish general population [26]. The reason for this finding might be due to the fact that the specimens of our study come from a group of females who attended an outpatient gynecologic clinic in the course of follow-up of healed or persistent cervical pathology. The assessment of HPV genotype in relation to *ab initio *cytologic diagnoses is beyond the scope of our study. The overall percentage (68.3%) of HPV positivity is within the range of positivity found in a Spanish study using linear arrays for genotyping in cytologic specimens from an outpatient gynecologic clinic (49.5%) [7,27]. The percentage of HPV positivity in HSIL found in our work (87.2%) is rather similar to that (90.8%) reported by Gonzalez-Bosquet et al. in a Spanish cohort of females with CIN2 and CIN3, the histologic counterparts of HSIL [7].

There are some similarities in the HPV genotype distribution found in N/B cases from the cohort studied compared to that reported by Gomez-Roman et al. which genotyped cytologic specimens from a cohort of females from northern Spain attending an outpatient gynecologic clinic; most frequent genotypes encountered were HPV16, 53 and 58. In our study HPV53 was also found to be the second most common genotype and HPV58 the third most prevalent high-risk genotype. HPV53, although considered as low-risk [28] or as probable high-risk genotype by others [25] does not seem to belong to the most prevalent HPV types reported worldwide in cervical specimens [25] though in some Italian studies HPV53 was found as the second (2.7%) and the fifth (3.6%) most prevalent genotype in the general female population and in females with abnormal smears [29,30]. In the study by Ortiz et al. HPV53 was found to be amongst the six most common genotypes in the general population from Madrid and Alicante [26] suggesting a higher prevalence of this genotype both in Spain and Italy. The combination of amplification and hybridization used in our study for all samples may overcome the lack of sensitivity of HPV53 detection found in other studies in which amplification with GP5+/6+ primers has been used as screening for HPV positive samples. This system has been shown to under-represent HPV53 as reported by Clifford et al. and Qu et al. [31,32]. Another reason for HPV53 under-representation in other studies is that HPV53 is detected but not genotyped due to lack of specific genotype probes when using the Hybrid Capture II current high-risk probe cocktail [33].

HPV58 seems to be most frequently found than HPV18 in N/B cases. A similar finding is reported elsewhere in other regions of Spain [29,34]. This considerable high prevalence of HPV58 in N/B cases from our region is particularly relevant since, as preliminary results by the PATRICIA study group have revealed that, unlike genotypes 31, 33, 52, vaccination against HPV16 and 18 does not seem to cross-protect against HPV58 [12]. This genotype is not as frequently found as in similar cohorts of females from Italy and France [29,30,35].

In LSIL cases HPV51 and HPV53 were, along with HPV31 and 33, the most frequent genotypes detected after HPV16. Other studies from Spain and France have also reported a high prevalence of HPV51 and 53 in LSIL cases [7,34].

In our study the most common genotypes found in HSIL were HPV16, 45, 51, 33 and 31. HPV16, 51, 33 and 31 are amongst the five most frequent genotypes found in HSIL specimens in previous studies from Spain and France [7,36]. Surprisingly, HPV45 is the second most common genotype in our HSIL cases, whilst its prevalence is lower in N/B and LSIL cases. The aforementioned studies did not find HPV45 to be one of the most prevalent types in HSIL though this type has been associated with this lesion. Though a possible overestimation of this genotype might have occurred due to the limited number of HSIL, HPV45 have been associated with HSIL in several studies [37].

As seen in Figure 1 HPV16 prevalence is increasing with the grade of lesion (p < 0.005). This finding underlines the higher oncogenic potential of HPV16 genotype compared to the other frequent genotypes found in our study such as 58, 31, 33 and 45. In fact, according to the seminal epidemiologic study by Muñoz et al., women infected with HPV16 have 434.5 fold higher risk to develop squamous cervical cancer than women HPV-negative. This risk decreases to 373.5, 248.1, 200.0, 197.6, 123.6, 114.8, 66.5, 45.1 and 4.3 for the genotypes 33, 18, 52, 45, 31, 58, 51, 56 and 6 respectively [25]. It is also worth mentioning that based on our data 34.6% of LSIL and 35.8% of HSIL would be prevented by HPV vaccination. However, these lesions would not necessary lead to invasive cervical cancer and the real impact of these vaccines will have to be evaluated in the future by the reduction of HPV16 and HPV18 cases in terms of incidence and mortality. The hypothetical protection provided by HPV vaccination in our region would be similar to that reported by González-Bosquet et al. in Barcelona. However these estimations do not consider a possible cross-protection effect that may enhance the vaccine effectiveness [7].

In our work the presence of co-infection was observed in 15.3% of N/B cases, 10.7% of ASC-US, 48.0% of LSIL and 25.6% of HSIL. These differences in co-infection occurrence are statistically significant (p < 0.0001). Rousseau et al. also reported an increased frequency of co-infection from N/B cases (3%) to ASC-US (10%) and to LSIL (23%) and a decreased frequency from LSIL to HSIL (7%) [8]. General lower prevalence of co-infection in the latter study might be due to the fact that the population included had not been selected for any type of gynaecologic pathology. The percentage of both HPV positivity and co-infection found in ASC-US suggests an HPV profile more similar to N/B than LSIL.

Our results suggest that LSIL may constitute a permissive state where the presence of certain genotypes as HPV16 may pave the way for other more co-infection-dependent genotypes, but as the lesion progresses only highly oncogenic types (i.e. HPV16) may persist whereas less oncogenic genotypes (i.e. HPV58, HPV53 and HPV6) are cleared off.

The greater number of coinfecting genotypes associated with their lower frequency suggests that the acquisition of two genotypes do not favour subsequent HPV types acquisition. According to our data it seems that genotypes from certain species (A5, A7, A9) appear as coinfecting more frequently than genotypes from other species (A6). This finding might indicate that there are different grades of dependency for each genotype or species to colonize cervical epithelium. The strength of the association and whether this probable dependency is intra or interspecies must be clarified in further studies. To the best of our knowledge this observation has not been previously reported. These results give support to the theory that the elimination of certain genotypes by vaccination may affect the distribution of other genotypes.

Therefore, based on our results we hypothesize that the reason why HPV vaccine does not show cross-protection against HPV58 as shown in the PATRICIA study [12] may perhaps be caused by its lack of co-infection dependency, whilst vaccine does show this cross-protection effect against other genotypes from the same A9 species (HPV31, 33, 52) which are more frequently found as coinfecting. It is worth mentioning that results from PATRICIA study concerning cross-protection aspects are still preliminary and have not necessarily proven to bear clinical significance.

## Conclusion

In this study we show that coinfecting genotype distribution and its prevalence in different grade of lesions suggests probable genotype interplay in the cervical epithelium. Simultaneous synergy or competing relationship amongst them cannot be ruled out. Therefore the vaccination-driven elimination of one HPV type would affect the natural history of the rest of genotypes and subsequently that of cervical carcinoma and would reinforce the necessity of a constant survey of genotypes in any geographical area where the HPV vaccination campaign is being implemented.

## Competing interests

The authors declare that they have no competing interests.

All authors state that the study sponsors (Fundación para la Formación e Investigación Sanitarias and Fundación CajaMurcia) have not been involved in either the study, design, the data collection, analysis and interpretation or the writing of the manuscript.

## Authors' contributions

PCZ and MEC designed experiments, SOR and MPG performed pathological diagnoses, ADP and JMB performed genotyping, FJOC performed statistical analysis. PCZ and MEC wrote grant applications and draft. Draft was corrected and approved by all authors.

## Pre-publication history

The pre-publication history for this paper can be accessed here:

http://www.biomedcentral.com/1471-2334/9/124/prepub
